# Effects of Different Isolation Media on Structural and Functional Properties of Starches from Root Tubers of Purple, Yellow and White Sweet Potatoes

**DOI:** 10.3390/molecules23092135

**Published:** 2018-08-24

**Authors:** Ahui Xu, Ke Guo, Tianxiang Liu, Xiaofeng Bian, Long Zhang, Cunxu Wei

**Affiliations:** 1 Key Laboratory of Crop Genetics and Physiology of Jiangsu Province/Key Laboratory of Plant Functional Genomics of the Ministry of Education, Yangzhou University, Yangzhou 225009, China; 15705271767@163.com (A.X.); 18115657147@163.com (K.G.); tianxiangliu1993@163.com (T.L.); zhanglong@yzu.edu.cn (L.Z.); 2 Co-Innovation Center for Modern Production Technology of Grain Crops of Jiangsu Province/Joint International Research Laboratory of Agriculture & Agri-Product Safety of the Ministry of Education, Yangzhou University, Yangzhou 225009, China; 3 Institute of Food Crops, Jiangsu Academy of Agricultural Sciences, Nanjing 210014, China; bianxiaofeng2@163.com

**Keywords:** sweet potato, starch, isolation medium, structural properties, functional properties

## Abstract

Different-colored sweet potatoes have different contents of pigments and phenolic compounds in their root tubers, which influence the isolation of starch. It is important to justify the identification of the most suitable isolation medium of starch from different colored root tubers. In this study, starches were isolated from root tubers of purple, yellow and white sweet potatoes using four different extraction media, including H_2_O, 0.5% Na_2_S_2_O_5_, 0.2% NaOH, and both 0.5% Na_2_S_2_O_5_ and 0.2% NaOH. Their structural and functional properties were investigated and compared among different extraction media. The results showed that the granule size, apparent amylose content, lamellar peak intensity, thermal properties, and pasting properties were different among different-colored sweet potatoes due to their different genotype backgrounds. The four extraction media had no significant effects on starch structural properties, including apparent amylose content, crystalline structure, ordered degree, and lamellar peak intensity, except that the NaOH and Na_2_S_2_O_5_ treatment were able to increase the whiteness of purple and yellow sweet potato starches. The different extraction media had some effects on starch functional properties, including thermal properties, swelling power, water solubility, and pasting properties. The above results indicated that the H_2_O was the most suitable extraction medium to simply and fast isolate starch from root tubers of different-colored sweet potatoes.

## 1. Introduction

Sweet potato [*Ipomoea batatas* (L.) Lam.] is one of the world’s important starch-producing crops. Its dry root tuber contains 50 to 80% starch, and is also rich in dietary fiber, vitamin C, provitamin A, iron, and minerals [1,2]. Three commonly available sweet potato varieties have purple, yellow and white root tubers due to the different contents of phenolic compounds and pigments in their root tubers [3,4,5]. The purple sweet potato has a deep purple color due to the accumulation of anthocyanin. The anthocyanin produced in cytosol, a group of water-soluble flavonoids, is transported into and stored in vacuoles [4]. The yellow sweet potato has a yellow color due to the accumulation of lipid-soluble β-carotene, which serves as the source of provitamin A showing vitamin A activity [5]. Compared with the purple and yellow sweet potatoes, the white sweet potato contains very low contents of phenolic compounds and β-carotene, but has no anthocyanins [3,5].

The root tuber of sweet potato is a good resource for starch [1]. The structural and functional properties of sweet potato starch, which determine the quality and application of starch in food and nonfood industries, have been studied extensively in white, yellow, orange, and purple sweet potatoes [6,7,8,9,10,11,12,13]. The Arracacha starches from white, yellow, and purple tubers have similar morphologies, but show significant differences in some physicochemical properties [14]. The different contents of pigments and phenolic compounds in root tubers influence starch isolation, especially the color of starch. In the previous literature, starch is isolated from different colored root tubers of sweet potato using H_2_O extraction [2,6,7,8], 0.2% (*w*/*v*) NaOH extraction [9,10], 0.2% (*w*/*v*) Na_2_S_2_O_5_ extraction [11,12], and 0.1% (*w*/*v*) NaHSO_3_ extraction [13]. The approximate 0.5% (*w*/*v*) Na_2_S_2_O_5_ extraction is also used to isolate starch from maize endosperm [15]. The NaOH can effectively remove the surface protein of starch, and the Na_2_S_2_O_5_ and NaHSO_3_ treatment can effectively minimize browning of the sample during starch isolation [9,12,15].

Many studies have shown that extraction media affect the structural and functional properties of starch [16,17,18]. Based on our knowledge, the effects of isolation media on the structural and functional properties of starches from different colored root tubers are unclear. Different-colored sweet potatoes have different contents of phenolic compounds and pigments [3,4,5]. Therefore, it is important to justify identification of the most suitable isolation medium of starch from different colored root tubers.

In this study, starches were isolated from purple, yellow and white sweet potatoes using H_2_O, 0.5% Na_2_S_2_O_5_, 0.2% NaOH, and both 0.5% Na_2_S_2_O_5_ and 0.2% NaOH extraction media. Their structural and functional properties were investigated and compared. Our objectives were to evaluate the effects of extraction media on starch properties, and find a suitable medium for isolating starch from different-colored sweet potatoes. This study could provide an important reference for the isolation method of starch from different-colored sweet potatoes, and would be helpful in the applications of sweet potato starches in food and nonfood industries.

## 2. Results and Discussion

### 2.1. Color of Starch

The color values of isolated starches are presented in Table 1. The color is one physical property of starch, and the whiteness is an important criterion in evaluating starch quality [17]. For starches extracted with H_2_O, the color was significantly different among the three varieties. L*-value was the highest for white-fleshed sweet potato and the lowest for purple-fleshed sweet potato (Table 1). The reason for the color differences among the purple-, yellow-, and white-fleshed sweet potatoes was due to the browning reactions during grinding of tubers [9]. The whiteness was the lowest for starch extracted with H_2_O, and was similar between both starches extracted with Na_2_S_2_O_5_ and NaOH, indicating that both Na_2_S_2_O_5_ and NaOH could effectively decrease the browning reactions of tissue during starch isolation, and increase the whiteness of starch. Kim et al. [9] isolated sweet potato starch using NaOH extraction, but without anhydrous ethanol treatment. There are some lipid-soluble pigments, such as β-carotene, in root tubers of white, yellow and purple sweet potatoes [4,5]. The lipid-soluble pigments cannot be removed through H_2_O, NaOH and Na_2_S_2_O_5_ washing during starch isolation. Therefore, the anhydrous ethanol washing must be performed to remove the lipid-soluble pigments. In the present study, the starch was washed with anhydrous ethanol many times until the supernatant was colorless. Therefore, the color of starch in the present study was whiter than that in Kim et al. [9]. Compared with previous isolation methods [6,7,8,9,10,11,12,13], the present starch extraction protocol has the advantages of improving starch purity, decreasing pigment content, and increasing starch whiteness.

### 2.2. Protein Content in Starch

The protein in starch is normally classified into two types: granule surface and interior protein [19]. The surface protein is easily removed from starch using some mild extraction methods without destroying the granule structure, while the removal of interior protein requires more disruptive extraction methods [20]. The starches extracted with four media were measured for residual protein. The protein content in starch is presented in Table 1. In all cases, the remaining protein contents were less than 0.7 mg/g, and had no significant differences among the starches isolated with different extraction media. The Na_2_S_2_O_5_ and NaOH extraction media are usually used to remove the surface protein from starch granules of cereals with a high protein content [15,21]. In the present study, compared with H_2_O extraction, the Na_2_S_2_O_5_ and NaOH treatment did not significantly decrease the protein content in starch, which might be due to the very low protein content in sweet potato. Usually, root and tuber starches have a very low protein content [19].

### 2.3. Morphology and Granule Size of Starch

**Figure 1:**
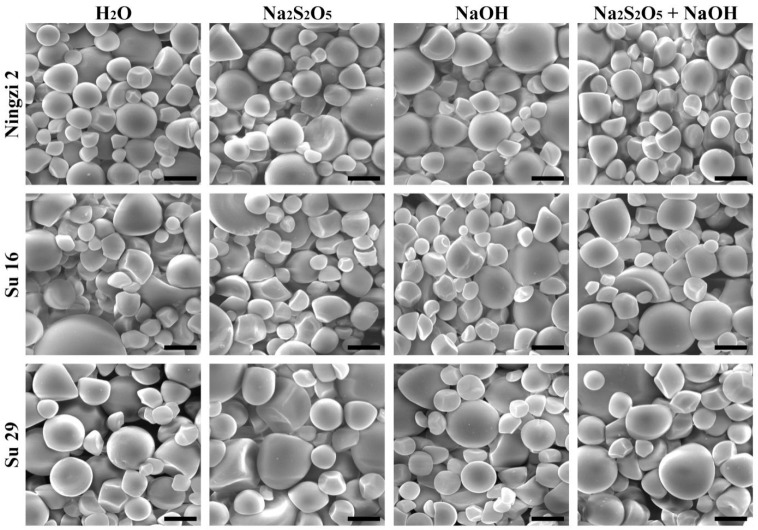
SEM photographs of starch granules. Scale bar = 10 μm.

**Figure 2:**
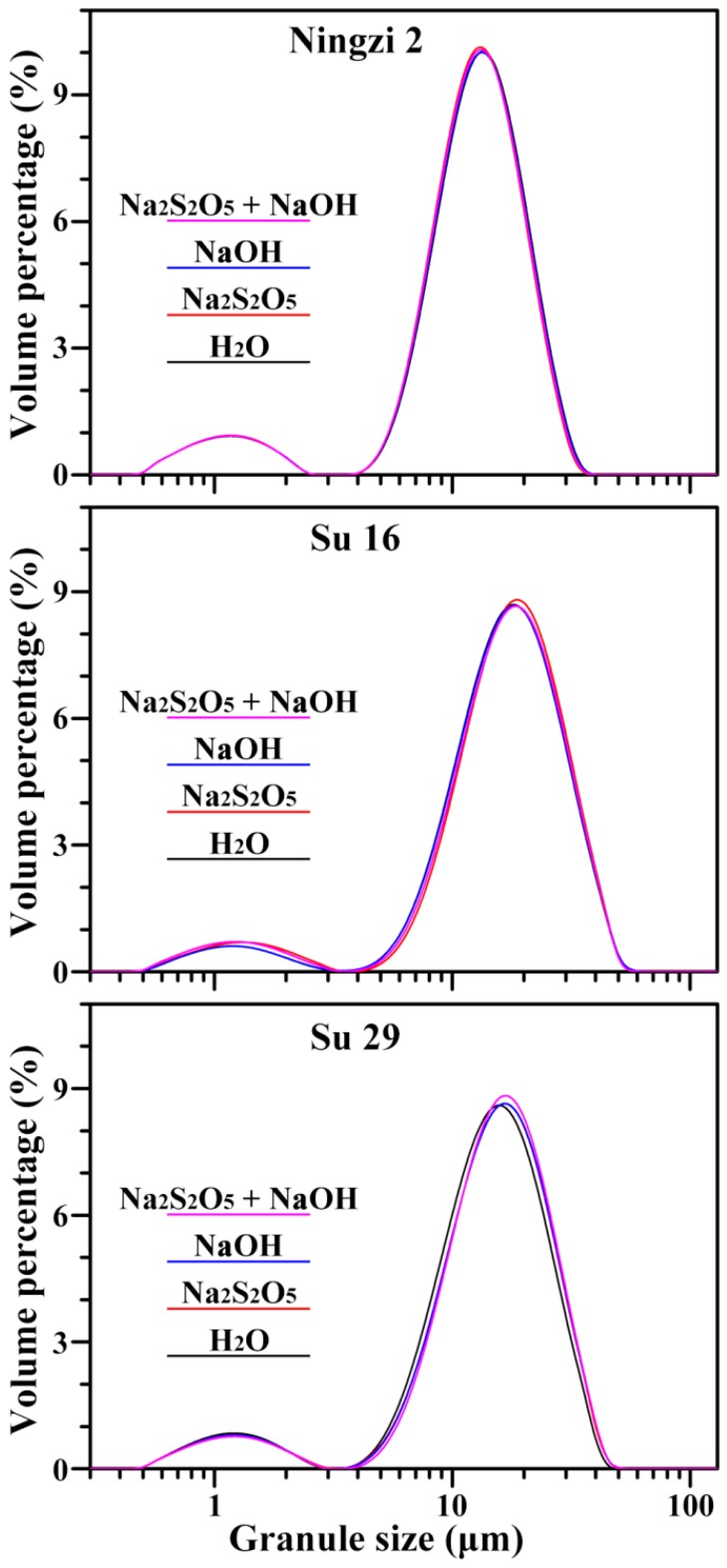
Size distribution of starch granules.

The starch granules from Ningzi 2, Su 16, and Su 29 were similar in morphology, exhibited round, polygonal, oval, and semi-oval shapes, and had large and small granules (Figure 1). These results were in agreement with the previous reports on different sweet potato varieties [6,9,10,12]. However, granule size distribution exhibited significant differences among the three varieties (Figure 2; Table 2). The volume-weighted mean diameters were 12.3, 17.2 and 15.1 μm for Ningzi 2, Su 16, and Su 29, respectively. These results were in agreement with previous reports on different sweet potato varieties, indicating that the difference in granule size might be due to different genotype backgrounds [22]. Na_2_S_2_O_5_ and NaOH treatment had no effect on starch morphology and size (Figure 1 and Figure 2, Table 2).

### 2.4. Iodine Absorption Spectrum and Apparent Amylose Content of Starch

**Figure 3:**
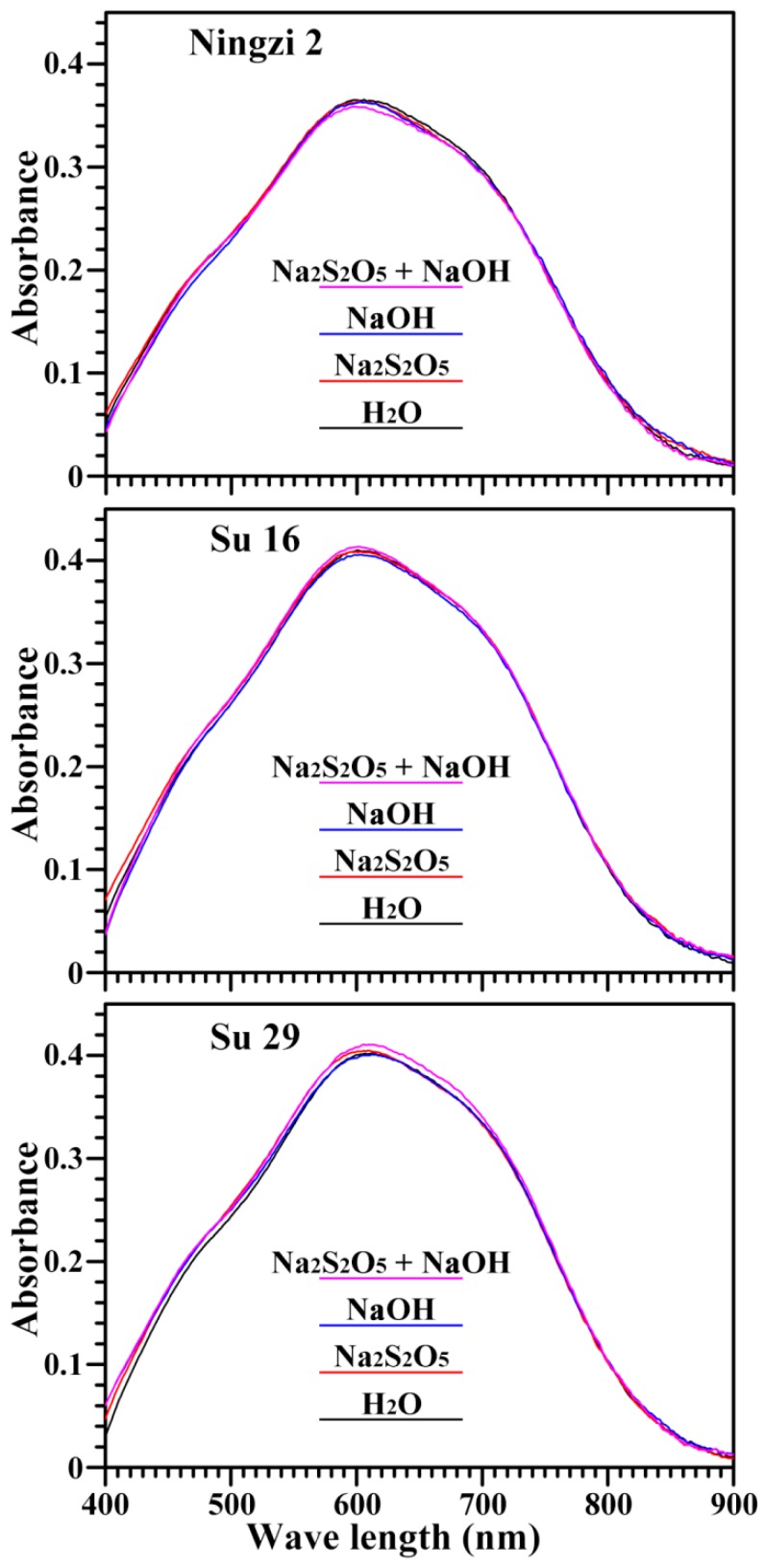
Spectra of iodine absorbance of starches.

The absorption spectrum of the starch-iodine complex is shown in Figure 3, and its derived maximum absorption wavelength (λ_max_), OD620/550 and apparent amylose content are presented in Table 3. The maximum absorption wavelength is related to the polymerization degree and average chain length of amylose and amylopectin; the OD620/550 reflects the relative content of longer chain segments in starch; and the apparent amylose content indicates the iodine absorbance from both amylose and longer branch-chains of amylopectin [23]. Na_2_S_2_O_5_ and NaOH treatment had no significant effect on the absorption spectrum of starch-iodine complex, including OD620/550 and apparent amylose content. No significant differences were also reported in the apparent amylose contents of starches isolated with different extraction media in acorn starches [16] and pea starches [18]. However, the apparent amylose content in Ningzi 2 starch was lower than that in Su 16 and Su 29 starches. The different apparent amylose content among different varieties resulted mainly from the different backgrounds of sweet potatoes. Some papers have reported that the apparent amylose contents range from 15.3 to 21.1% in two purple and two orange fleshed cultivars of Japanese sweet potatoes [24], and from 23.3 to 26.5% in two white and nine purple fleshed cultivars of Chinese sweet potatoes [2].

### 2.5. Crystalline Structure of Starch

**Figure 4:**
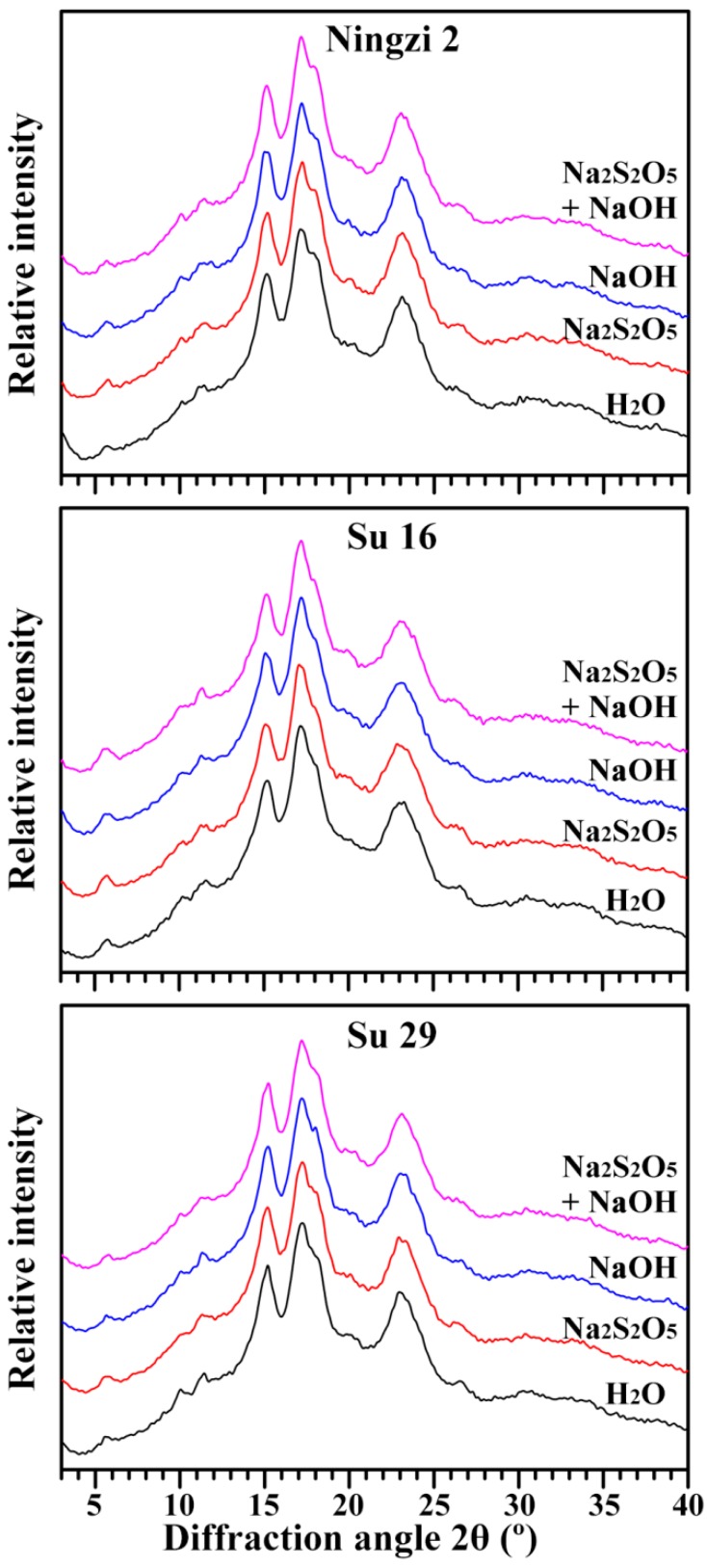
XRD patterns of starches.

The X-ray powder diffraction (XRD) patterns of starches are shown in Figure 4. Native starches from different plant sources can be divided into three types of A-, B-, and C-type according to their XRD patterns. C-type starch contains both A- and B-type crystallinities, and can be further divided into C_A_- (closer to A-type), C_C_- (typical C-type) and C_B_-type (closer to B-type) starches according to the proportion of A- and B-type crystallinity from high to low. The C_C_-type starch has strong diffraction peaks at about 17° and 23° 2θ, and small peaks at about 5.6° and 15° 2θ. The peak at 5.6° 2θ is the characteristic of B-type crystallinity. Compared with C_C_-type starch, the C_A_-type starch has a shoulder peak at about 18° 2θ, which is the characteristic peak of A-type crystallinity [25]. According to XRD patterns of starches extracted with H_2_O, the Ningzi 2, Su 16 and Su 29 starches all showed C_A_-type XRD patterns due to the existence of shoulder peak at 18° 2θ. It is noteworthy that the intensity of the shoulder peak at 18° 2θ was the weakest and that of the peak at 5.6° 2θ was the strongest in Su 16 starch, indicating that the proportion of B-type crystallinity was higher in Su 16 starch than in Ningzi 2 and Su 29 starches. The A-, C_A_-, C_B_- and C-type starches have been reported in different sweet potato varieties in previous papers [9,10,22,26]. Though the environment, especially growth temperature, has some effects on the crystalline structure in sweet potato, the different proportions of A- and B-type crystallinity in the present study might be due to their different genotypes, given that they grew in the same environment. Different extraction media of starch had no effect on XRD patterns of three sweet potato varieties (Figure 4). The relative crystallinities of H_2_O, Na_2_S_2_O_5_, NaOH, and both Na_2_S_2_O_5_ and NaOH treatment were 22.2%, 23.2%, 22.1%, and 23.3% for Ningzi 2, 20.8%, 21.5%, 21.2%, and 21.4% for Su 16, and 22.8%, 22.4%, 21.9%, and 22.2% for Su 29. These results showed that the Na_2_S_2_O_5_ and NaOH treatment had no effect on the starch crystalline structure.

### 2.6. Short-Range Ordered Structure of Starch

**Figure 5:**
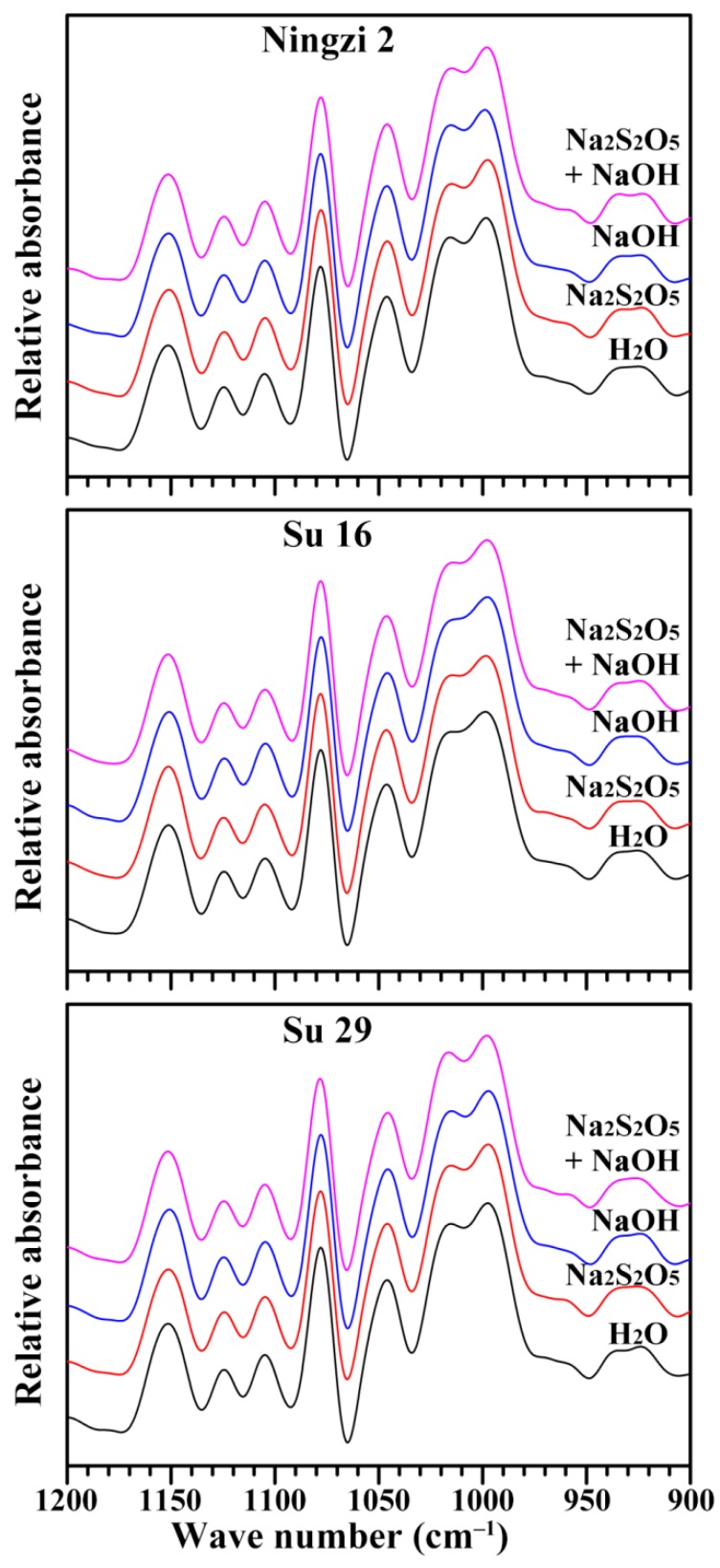
ATR-FTIR spectra of starches.

The short-range ordered structure of starch, defined as the double-helical order, can be detected using attenuated total reflectance-Fourier transform infrared (ATR-FTIR) spectrometer. The ATR-FTIR spectrum can reflect the short-ranged ordered structure in the starch external region [27]. The deconvoluted ATR-FTIR spectra of starches extracted using different media are presented in Figure 5. The spectra show bands at 1100–1050 cm^−1^ including at approximately 1150, 1125 and 1105 cm^−1^ (C–O, C–C and C–O–H stretching), and 1100–900 cm^−1^ including at approximately 1078, 1045, 1022, 995 and 925 cm^−1^ (C–O–H bending). The bands in the region 1100–900 cm^−1^ are sensitive to changes in starch structure; the bands at 1047 and 1022 cm^−1^, especially, are widely used to reflect the ordered structure [28]. The bands at 1045 and 1022 cm^−1^ are associated with ordered/crystalline and amorphous regions in starch, respectively. The ratio of absorbance 1045/1022 cm^−1^ can be used to quantify the ordered degree [27]. The starches extracted using different media had similar ATR-FTIR spectra (Figure 5). IR ratio of 1045/1022 cm^−1^ of H_2_O, Na_2_S_2_O_5_, NaOH, and both Na_2_S_2_O_5_ and NaOH treatment were 0.70, 0.70, 0.69, and 0.71 for Ningzi 2, 0.71, 0.70, 0.71, and 0.71 for Su 16, and 0.71, 0.70, 0.70, and 0.70 for Su 29. These results indicated that the Na_2_S_2_O_5_ and NaOH treatment had no effect on the double-helical order of starch in granule external region. Similar results are also reported in parota starches isolated using H_2_O, sodium bisulfite and acid steeping [17].

### 2.7. Lamellar Structure of Starch

**Figure 6:**
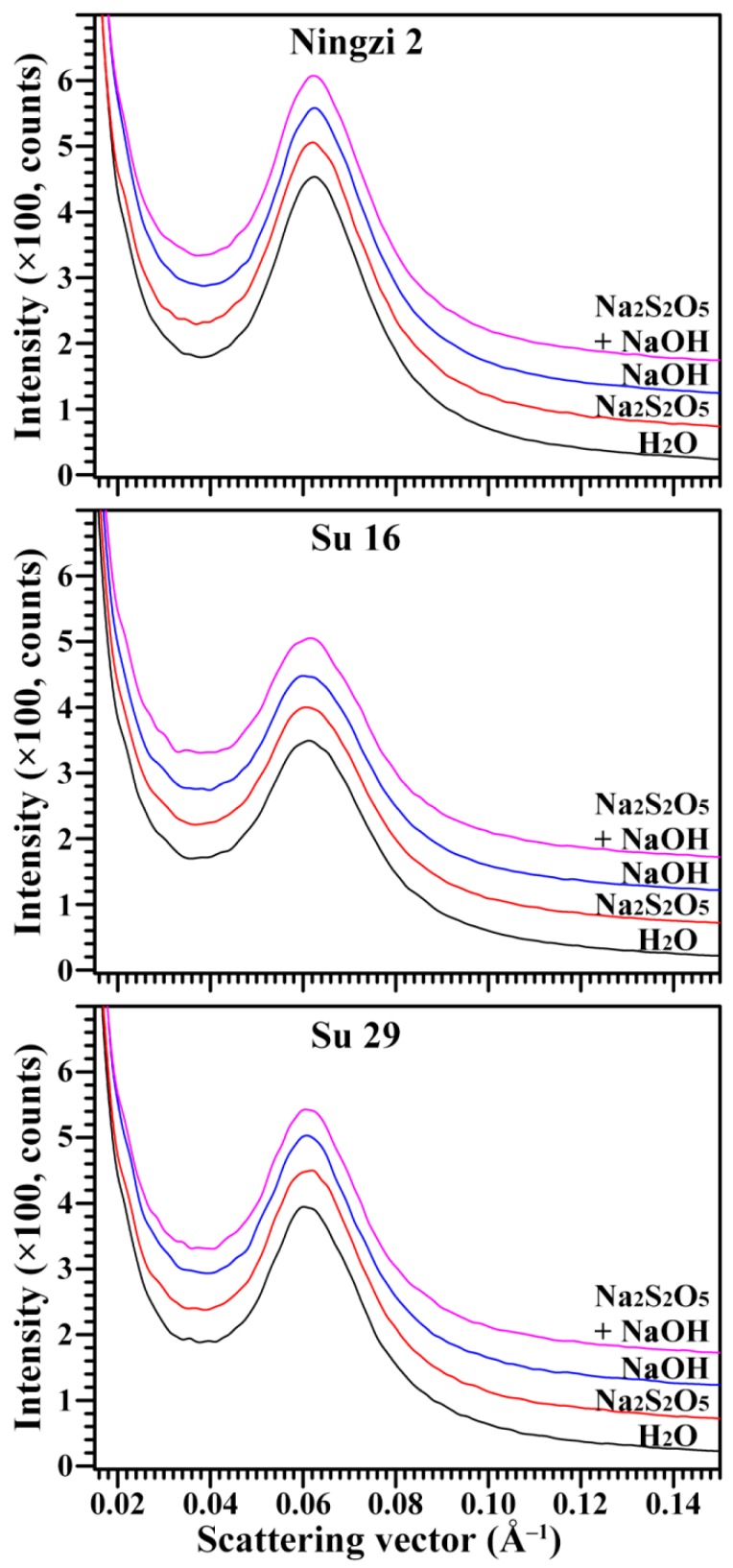
SAXS patterns of starches.

The lamellar structure of alternating amorphous and crystalline regions in starch granules can be detected by a small-angle X-ray scattering (SAXS) instrument [29]. The SAXS spectra of sweet potato starches with different extraction media are presented in Figure 6. All spectra were normalized to equal intensity at high q (q = 0.2 Å^−1^) to account for variations in sample concentration, leading to the spectra being at the same relative scale, and therefore directly comparable [30]. The parameters of the SAXS peak, including peak position (S_max_) and intensity, can be measured by a simple graphical method, and the lamellar repeat distance (D) can be calculated from the S_max_ according to D = 2π/S_max_ [31]. The amorphous region in starch contributes to the background of scattering peak, and the nanocrystalline lamellae contribute to the intensity and width of peak. The Ningzi 2, Su 16 and Su 29 starches had similar scattering peaks at 0.062 Å^−1^, corresponding to a lamellar repeat distance of approximate 10.2 nm. The different extraction media had no effect on lamellar repeat distance. However, the peak intensity was significantly different among the three sweet potato varieties, at about 320, 220, and 260 for Ningzi 2, Su 16, and Su 29, respectively. The peak intensity depends mainly on the degree of ordering in semicrystalline regions [29]. The Na_2_S_2_O_5_ and NaOH treatment had no effect on lamellar peak intensity.

### 2.8. Thermal Properties of Starch

**Figure 7:**
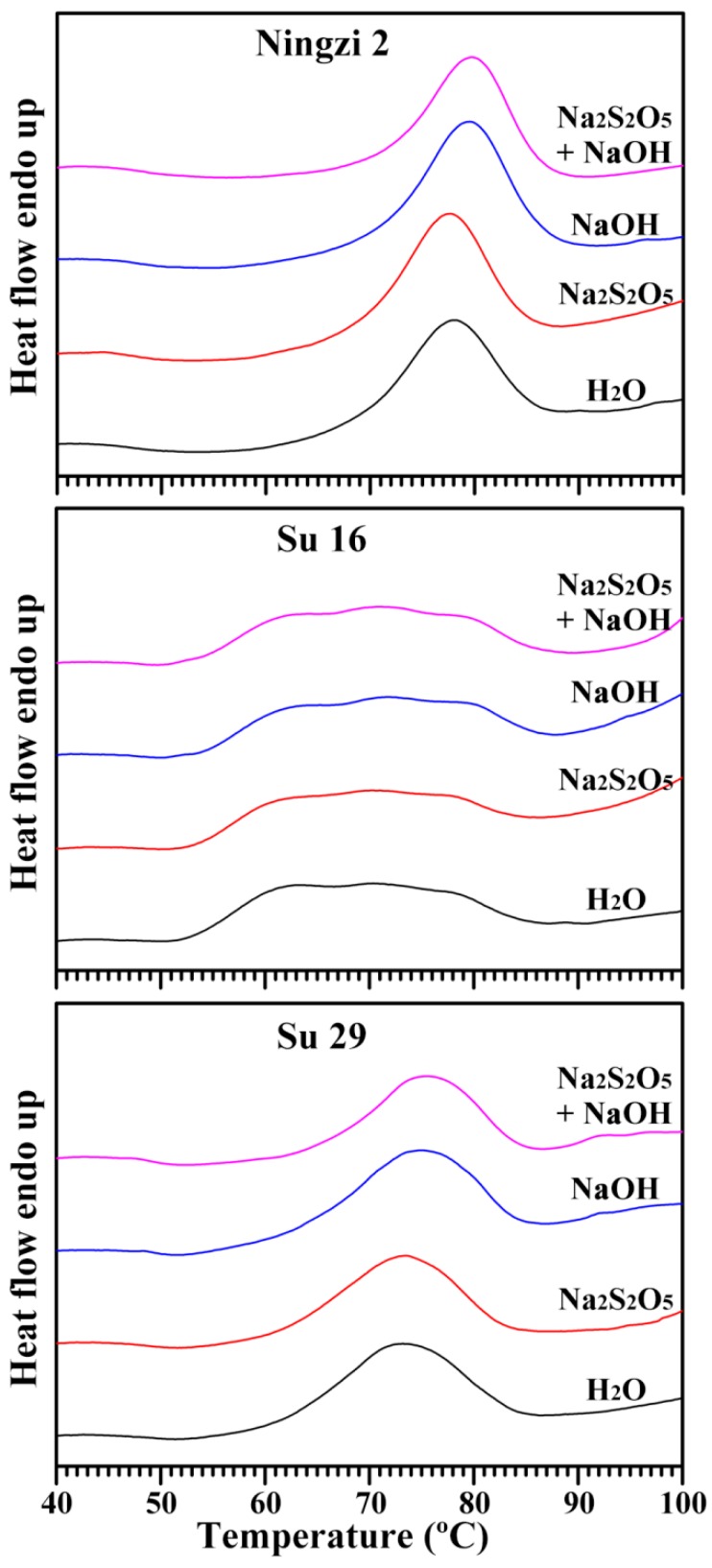
Differential scanning calorimetry thermograms of starches.

The thermal properties of sweet potato starches isolated using four extraction media are presented in Figure 7 and Table 4. For starch isolated using H_2_O extraction, significant differences were detected in the three sweet potato starches. Ningzi 2 starch had a single gelatinization peak with high gelatinization temperatures and enthalpy and low gelatinization temperature range, and Su 16 starch had a wide gelatinization peak with low onset gelatinization temperature and wide gelatinization temperature range. Similar results have also been reported in some sweet potato starches [7,22]. In the present study, the different thermal properties might be due to the different proportions of A- and B-type crystallinity in C-type starch (Figure 4). Usually, A-type crystallinity has a high gelatinization temperature and B-type crystallinity has a low gelatinization temperature [32]. For the same sweet potato, starches isolated with different extraction media had slightly differences in their gelatinization temperatures and enthalpy though their thermograms were similar. The thermal properties of starch are related to a variety of factors, including granule morphology and size, amylose content, crystalline structure, and protein and lipid contents [33].

### 2.9. Swelling Power and Water Solubility of Starch

The swelling powers and water solubilities of starches at 95 °C are shown in Table 5. The swelling power is a measure of the water-holding capacity of starch after being heated, cooled, and centrifuged, while the water solubility reflects the degree of dissolution during the starch swelling procedure [22]. The hydration and swelling of starch during heating reflects the magnitude of interaction between starch chains. The different sweet potato starches had different swelling power and water solubility, but starches isolated from the same sweet potato with different extraction media had similar swelling power and water solubility, indicating that NaOH and Na_2_S_2_O_5_ extraction had an effect on the swelling power and water solubility of starch. Similar results have also been reported in acorn starches isolated with alkaline and enzymatic extraction media [16].

### 2.10. Pasting Properties of Starch

**Figure 8:**
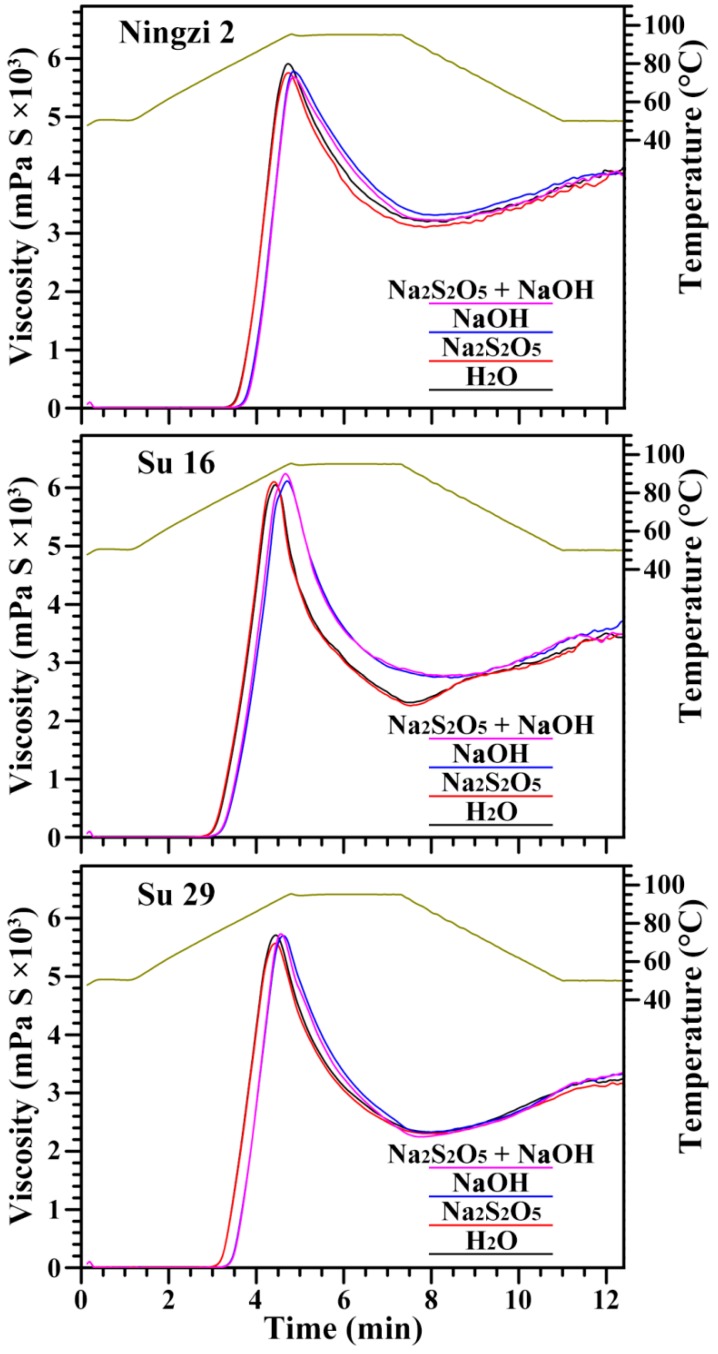
Pasting profiles of starches.

The pasting properties of starches, an important functional property determining the quality and utilization of starch, were analyzed using a rapid visco analyzer. The changes in pasting profile are influenced by structural, thermal, and morphological changes taking place as a result of temperature [34]. The pasting profiles of starches are shown in Figure 8, and the pasting parameters are presented in Table 6. Peak viscosity reflects the ability of starch to bind water via hydrogen bonds, and final viscosity indicates the stability to the swollen granule structure. Breakdown viscosity can evaluate the starch pasting resistance to heat with lower value having higher ability to withstand heating, and setback viscosity reflects the tendency of starch paste to retrogradation. Pasting temperature can reflect the energy cost required during cooking [13,22]. The significantly different pasting viscosities and temperatures were detected in the three sweet potato starches, which might be due to the different granule size (Figure 2), apparent amylose content (Table 3), proportion of A- and B-type crystallinity (Figure 4), or lamellar peak intensity (Figure 6) [9,22]. The different extraction media had some effect on the pasting viscosities and temperatures of starch (Table 6). Similar phenomena have also been reported in pea starch isolated with dry-milling, sour liquid processing, alkaline steeping and neutral protease extraction methods [18], and in rice starches isolated with neutral protease and alkaline steeping methods [35].

## 3. Materials and Methods

### 3.1. Plant Materials

Purple sweet potato variety Ningzi 2, yellow sweet potato variety Su 16, and white sweet potato variety Su 29 were used in this study. They were grown under normal agronomic practices in the experimental field of Jiangsu Academy of Agricultural Sciences, Nanjing, China in 2017. The root tubers after harvest were stored in dark with about 85% humidity at 12 °C for about one month before use.

### 3.2. Starch Isolation

**Figure 9:**
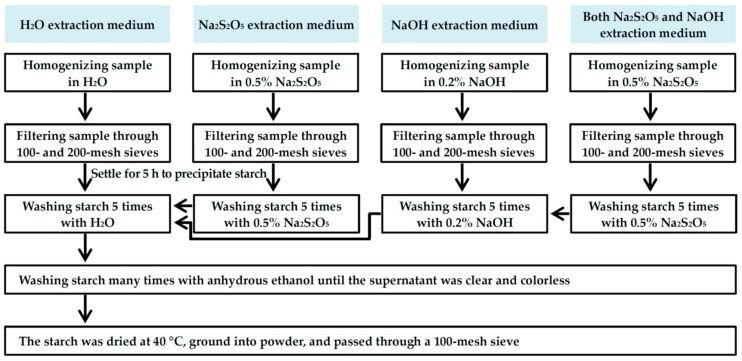
Procedure for starch isolation from root tubers with different extraction media.

Starch was isolated from the root tubers using the following four extraction media according to the methods of Lai et al. [8], Osundahunsi et al. [11], and Kim et al. [9], with many modifications. Briefly, the root tubers were washed and cut into small pieces. The starch was isolated from sample pieces using four different extraction media, and their procedures are shown in Figure 9. The samples were homogenized in extraction medium using a home blender.

### 3.3. Color Evaluation of Starch

The color of starch was measured using a portable colorimeter (CR-400, Konicca Minolta Sening Inc., Osaka, Japan) in which L*, a*, and b* values were measured. The L*, a*, and b* values state the position on the white/black, red/green, and yellow/blue axis, respectively.

### 3.4. Measurement of Protein Content in Starch

The nitrogen content in dry starch was measured using an elemental analyzer (Vario EL cube, Elementar Analysensysteme Gmbh, Hanau, Germany), and then converted to protein content using a 6.25 conversion factor.

### 3.5. Morphology Observation and Granule Size Analysis of Starch

Starch powder was directly mounted on an aluminum stub using double-sided adhesive tape. The sample was coated with gold using a sputter coater and observed using an environmental scanning electron microscope (XL-30, Philips, Eindhoven, Holland). Starch granule size was analyzed using a laser diffraction particle size analyzer (Mastersizer 2000, Malvern, Worcestershire, UK) following the methods of Zhang et al. [22]. Briefly, starch was suspended in distilled water and stirred at 2000 rpm. The obscuration in all measurement was >10%.

### 3.6. Measurements of Iodine Absorption Spectrum and Apparent Amylose Content

The starch-iodine absorption spectrum and apparent amylose content were measured as previously described by Zhang et al. [22]. Briefly, starch was dissolved in urea dimethyl sulphoxide (UDMSO) solution and treated with iodine solution. The iodine absorption spectrum was scanned from 400 to 900 nm with a spectrophotometer (Ultrospec 6300 pro, Amersham Biosciences, Cambridge, Sweden). Apparent amylose content was evaluated from absorbance at 620 nm.

### 3.7. Crystalline Structure Analysis

The crystalline structure of starch was analyzed using an X-ray powder diffractometer (D8, Bruker, Karlsruhe, Germany). The signal-to-noise ratio of XRD spectrum is influenced by water content of starch [36]. The sample preparation, test condition setting, and measurement of relative crystallinity were described previously by Wei et al. [37]. Briefly, all the starch samples were stored in a desiccator, where a saturated solution of NaCl maintained a constant humidity atmosphere (relative humidity (RH) = 75%) for 1 week at 25 °C before measurement. The starch was scanned from 3° to 40° 2θ with a step size of 0.02° using the X-ray beam at 200 mA and 40 kV.

### 3.8. Short-Range Ordered Structure Analysis

The short-range ordered structure of starch was analyzed using a FTIR spectrometer (7000, Varian, Santa Clara, CA, USA) with a DTGS detector equipped with an ATR cell. The sample preparation and test condition setting were described previously by Wei et al. [37]. The original spectrum was corrected by subtraction of the baseline in the region from 1200 to 800 cm^−1^ before deconvolution. For deconvolution, the assumed line shape was Lorentzian, with a half-width of 19 cm^−1^ and a resolution enhancement factor of 1.9.

### 3.9. Lamellar Structure Analysis

The lamellar structure of starch was analyzed using a SAXS instrument (NanoStar, Bruker, Karlsruhe, Germany) equipped with Vantec 2000 detector and pin-hole collimation for point focus geometry. The sample preparation, test condition setting, and lamellar parameter analysis were described previously by Cai et al. [31]. Briefly, starch-water slurry was kept in a sealed cell. The X-ray source was a copper rotating anode (0.1 mm filament) operating at 50 kV and 30 W, fitted with cross-coupled Göbel mirrors, resulting in a Cu Kα radiation wavelength of 1.5406 Å. The optics and sample chamber were under vacuum to minimize air scattering.

### 3.10. Measurement of Thermal Properties

The thermal properties of starch were measured using a differential scanning calorimetry (200-F3, Netzsch, Selb, Germany) following the method of Zhang et al. [22]. Briefly, 5 mg of starch and 15 μL of distilled water were mixed and sealed in an aluminum pan, held at 4 °C overnight, and equilibrated for 2 h at room temperature before analysis. The samples were heated from 25 to 130 °C at a rate of 10 °C/min.

### 3.11. Measurement of Swelling Power and Water Solubility

The swelling power and water solubility of starch were measured at 95 °C as previously described by Lin et al. [15]. Briefly, 2% (*w*/*v*) starch-water slurry was heated in a water bath for 30 min, cooled to room temperature, and centrifuged (8000 *g*, 10 min). The supernatant was removed to measure the soluble carbohydrate using anthrone-H_2_SO_4_ method, and the precipitate was weighed to calculate the swelling power. The swelling power was the ratio of precipitate weight and starch weight after subtraction of the soluble carbohydrate weight, the water solubility was the amount of soluble carbohydrate by the original starch.

### 3.12. Measurement of Pasting Properties

The pasting properties of starch were measured using a rapid visco analyzer (RVA-3D, Newport Scientific, Warriewood, Australia) following the method of Zhang et al. [22]. Briefly, 8% starch-water slurry was dispersed by rotating the paddle at 960 rpm for the first 10 s and then at a constant speed of 160 rpm during analysis. The starch suspension was first held at 50 °C for 1 min, heated to 95 °C at a rate of 12 °C/min, held at 95 °C for 2.5 min, cooled down to 50 °C at a rate of 12 °C/min, and held at 50 °C for 1.4 min.

### 3.13. Statistical Analysis

The data reported in all the tables are means ± standard deviation. One-way analysis of variance by Tukey’s test was evaluated using the SPSS 16.0 Statistical Software Program.

## 4. Conclusions

In conclusion, starches were isolated from root tubers of purple, yellow and white sweet potatoes using H_2_O, 0.5% Na_2_S_2_O_5_, 0.2% NaOH, and both 0.5% Na_2_S_2_O_5_ and 0.2% NaOH extraction media. Starches from different colored root tubers had differences in granule size, amylose content, thermal properties, swelling power, water solubility, and pasting properties due to their different genotypes. Na_2_S_2_O_5_ and NaOH extraction were able to increase the whiteness of starch from purple and yellow sweet potato, but had no significant effect on apparent amylose content, crystalline structure, ordered degree, and lamellar structure of starches. The different extraction media had a slight effect on the thermal properties, swelling power, water solubility, and pasting properties of starches. This study indicates that H_2_O is the most suitable extraction medium for simply and quickly isolating starch from root tubers of different-colored sweet potatoes.

## Figures and Tables

**Table 1:** Color parameters and protein content (PC) of starches.

	L*	a*	b*	PC (mg/g)
Ningzi 2				
H_2_O	93.76 ± 0.16 ^a^	−0.10 ± 0.00 ^d^	+3.99 ± 0.04 ^b^	0.69 ± 0.00 ^a^
Na_2_S_2_O_5_	94.71 ± 0.17 ^b^	−0.22 ± 0.01 ^c^	+3.20 ± 0.03 ^a^	0.69 ± 0.00 ^a^
NaOH	94.70 ± 0.28 ^b^	−0.65 ± 0.02 ^a^	+3.21 ± 0.06 ^a^	0.59 ± 0.04 ^a^
Na_2_S_2_O_5_ + NaOH	95.20 ± 0.03 ^c^	−0.60 ± 0.01 ^b^	+3.20 ± 0.08 ^a^	0.59 ± 0.04 ^a^
Su 16				
H_2_O	95.45 ± 0.38 ^a^	−0.29 ± 0.02 ^c^	+1.74 ± 0.07 ^b^	0.56 ± 0.09 ^a^
Na_2_S_2_O_5_	96.37 ± 0.02 ^b^	−0.23 ± 0.01 ^d^	+1.41 ± 0.07 ^a^	0.59 ± 0.04 ^a^
NaOH	96.24 ± 0.23 ^b^	−0.57 ± 0.01 ^a^	+2.36 ± 0.03 ^d^	0.59 ± 0.04 ^a^
Na_2_S_2_O_5_ + NaOH	96.21 ± 0.17 ^b^	−0.41 ± 0.01 ^b^	+2.00 ± 0.03 ^c^	0.56 ± 0.09 ^a^
Su 29				
H_2_O	96.31 ± 0.22 ^a^	−0.24 ± 0.01 ^c^	+1.95 ± 0.06 ^d^	0.72 ± 0.13 ^a^
Na_2_S_2_O_5_	96.32 ± 0.26 ^a^	−0.10 ± 0.01 ^d^	+0.96 ± 0.04 ^a^	0.66 ± 0.04 ^a^
NaOH	96.41 ± 0.28 ^a^	−0.39 ± 0.01 ^a^	+1.64 ± 0.05 ^c^	0.56 ± 0.00 ^a^
Na_2_S_2_O_5_ + NaOH	96.87 ± 0.05 ^a^	−0.33 ± 0.01 ^b^	+1.24 ± 0.05 ^b^	0.56 ± 0.00 ^a^

L* = lightness value, 100 = white and 0 = black; a* = red/green, + for red and − for green; b* = yellow/blue, + for yellow and − for blue. Data are means ± standard deviations, *n* = 3. Values in the same column and variety with different superscript letters are significantly different (*p* < 0.05).

**Table 2:** Granule size of starches.

	d(0.1) (μm)	d(0.5) (μm)	d(0.9) (μm)	D[4, 3] (μm)
Ningzi 2				
H_2_O	5.517 ± 0.001 ^d^	11.937 ± 0.004 ^d^	20.294 ± 0.014 ^d^	12.361 ± 0.006 ^d^
Na_2_S_2_O_5_	5.443 ± 0.002 ^a^	11.662 ± 0.002 ^a^	19.733 ± 0.004 ^a^	12.056 ± 0.002 ^a^
NaOH	5.457 ± 0.001 ^c^	11.830 ± 0.002 ^c^	20.138 ± 0.005 ^c^	12.254 ± 0.002 ^c^
Na_2_S_2_O_5_ + NaOH	5.452 ± 0.001 ^b^	11.726 ± 0.003 ^b^	19.901 ± 0.004 ^b^	12.136 ± 0.003 ^b^
Su 16				
H_2_O	7.214 ± 0.002 ^c^	15.960 ± 0.004 ^a^	29.420 ± 0.017 ^a^	17.138 ± 0.008 ^a^
Na_2_S_2_O_5_	7.135 ± 0.003 ^b^	16.395 ± 0.007 ^d^	29.871 ± 0.021 ^d^	17.422 ± 0.009 ^d^
NaOH	7.228 ± 0.002 ^d^	16.038 ± 0.003 ^b^	29.612 ± 0.012 ^b^	17.234 ± 0.005 ^c^
Na_2_S_2_O_5_ + NaOH	6.869 ± 0.000 ^a^	16.126 ± 0.002 ^c^	29.653 ± 0.012 ^c^	17.181 ± 0.004 ^b^
Su 29				
H_2_O	5.654 ± 0.005 ^a^	13.606 ± 0.008 ^a^	25.039 ± 0.019 ^a^	14.472 ± 0.009 ^a^
Na_2_S_2_O_5_	5.881 ± 0.016 ^b^	14.254 ± 0.001 ^b^	26.072 ± 0.009 ^b^	15.117 ± 0.002 ^b^
NaOH	5.957 ± 0.001 ^c^	14.304 ± 0.002 ^c^	26.176 ± 0.011 ^c^	15.182 ± 0.004 ^c^
Na_2_S_2_O_5_ + NaOH	6.291 ± 0.003 ^d^	14.514 ± 0.004 ^d^	26.278 ± 0.001 ^d^	15.371 ± 0.002 ^d^

The d(0.1), d(0.5) and d(0.9) are the granule size at which 10%, 50% and 90% of all the granules by volume are smaller, respectively. The D[4, 3] is the volume-weighted mean diameter. Data are means ± standard deviations, *n* = 3. Values in the same column and variety with different superscript letters are significantly different (*p* < 0.05).

**Table 3:** Maximum absorption wavelength (λ_max_), iodine absorbance ratios of OD620 to OD550 (OD620/550), and apparent amylose contents (AAC) of starches.

	λ_max_ (nm)	OD620/550	AAC (%)
Ningzi 2			
H_2_O	607 ± 1 ^b^	1.14 ± 0.02 ^a^	26.0 ± 0.4 ^a^
Na_2_S_2_O_5_	603 ± 1 ^ab^	1.14 ± 0.01 ^a^	25.9 ± 0.9 ^a^
NaOH	604 ± 1 ^ab^	1.14 ± 0.01 ^a^	25.7 ± 0.8 ^a^
Na_2_S_2_O_5_ + NaOH	602 ± 2 ^a^	1.13 ± 0.00 ^a^	25.7 ± 0.4 ^a^
Su 16			
H_2_O	603 ± 1 ^a^	1.13 ± 0.01 ^a^	29.4 ± 0.7 ^a^
Na_2_S_2_O_5_	602 ± 2 ^a^	1.12 ± 0.02 ^a^	29.0 ± 1.2 ^a^
NaOH	605 ± 2 ^a^	1.14 ± 0.01 ^a^	28.3 ± 0.7 ^a^
Na_2_S_2_O_5_ + NaOH	603 ± 1 ^a^	1.13 ± 0.00 ^a^	29.8 ± 0.7 ^a^
Su 29			
H_2_O	611 ± 1 ^b^	1.19 ± 0.02 ^a^	29.2 ± 0.8 ^a^
Na_2_S_2_O_5_	608 ± 1 ^a^	1.17 ± 0.00 ^a^	30.2 ± 0.6 ^a^
NaOH	609 ± 1 ^ab^	1.17 ± 0.01 ^a^	28.8 ± 0.8 ^a^
Na_2_S_2_O_5_ + NaOH	611 ± 1 ^b^	1.19 ± 0.01 ^a^	28.8 ± 1.0 ^a^

Data are means ± standard deviations, *n* = 3. Values in the same column and variety with different superscript letters are significantly different (*p* < 0.05).

**Table 4:** Thermal properties of starches.

	To (°C)	Tp (°C)	Tc (°C)	ΔTo (°C)	ΔH (J/g)
Ningzi 2					
H_2_O	68.9 ± 0.3 ^a^	78.0 ± 0.1 ^a^	85.5 ± 0.4 ^a^	16.6 ± 0.7 ^a^	11.3 ± 0.5 ^a^
Na_2_S_2_O_5_	68.5 ± 0.1 ^a^	77.7 ± 0.1 ^a^	85.1 ± 0.4 ^a^	16.6 ± 0.5 ^a^	12.1 ± 0.4 ^ab^
NaOH	70.0 ± 0.4 ^b^	79.5 ± 0.1 ^b^	86.6 ± 0.3 ^b^	16.7 ± 0.1 ^a^	13.1 ± 0.0 ^b^
Na_2_S_2_O_5_ + NaOH	70.4 ± 0.1 ^b^	79.6 ± 0.2 ^b^	86.9 ± 0.5 ^b^	16.5 ± 0.6 ^a^	13.4 ± 0.1 ^b^
Su 16					
H_2_O	54.0 ± 0.1 ^c^	70.3 ± 0.1 ^a^	84.6 ± 0.1 ^ab^	30.7 ± 0.2 ^a^	9.4 ± 0.1 ^a^
Na_2_S_2_O_5_	53.3 ± 0.1 ^a^	70.1 ± 0.1 ^a^	83.9 ± 0.4 ^a^	30.6 ± 0.4 ^a^	9.2 ± 0.1 ^a^
NaOH	53.5 ± 0.0 ^ab^	71.7 ± 0.6 ^b^	86.4 ± 0.8 ^c^	32.9 ± 0.8 ^b^	10.4 ± 0.0 ^b^
Na_2_S_2_O_5_ + NaOH	53.6 ± 0.1 ^b^	71.1 ± 0.0 ^ab^	85.9 ± 0.2 ^bc^	32.3 ± 0.4 ^ab^	11.0 ± 0.3 ^c^
Su 29					
H_2_O	61.2 ± 0.1 ^a^	73.3 ± 0.0 ^a^	82.9 ± 0.6 ^a^	21.8 ± 0.6 ^a^	9.3 ± 0.1 ^a^
Na_2_S_2_O_5_	60.9 ± 0.1 ^a^	73.3 ± 0.1 ^a^	83.1 ± 0.1 ^a^	22.2 ± 0.0 ^a^	10.4 ± 0.1 ^b^
NaOH	62.4 ± 0.1 ^ab^	75.1 ± 0.1 ^b^	84.3 ± 0.1 ^a^	22.0 ± 0.2 ^a^	10.8 ± 0.0 ^b^
Na_2_S_2_O_5_ + NaOH	63.3 ± 0.9 ^b^	75.4 ± 0.3 ^b^	84.6 ± 0.9 ^a^	21.3 ± 0.0 ^a^	11.2 ± 0.6 ^b^

To, gelatinization onset temperature; Tp, gelatinization peak temperature; Tc, gelatinization conclusion temperature; ΔT, gelatinization temperature range (Tc − To); ΔH, gelatinization enthalpy. Data are means ± standard deviations, *n* = 3. Values in the same column and variety with different superscript letters are significantly different (*p* < 0.05).

**Table 5:** Swelling powers (SP) and water solubilities (WS) of starches.

	Ningzi 2		Su 16		Su 29	
	SP (g/g)	WS (%)	SP (g/g)	WS (%)	SP (g/g)	WS (%)
H_2_O	26.9 ± 0.3 ^ab^	11.1 ± 0.4 ^a^	32.4 ± 0.6 ^bc^	12.8 ± 0.4 ^a^	28.2 ± 0.1 ^a^	17.1 ± 0.2 ^b^
Na_2_S_2_O_5_	27.5 ± 0.3 ^b^	11.3 ± 0.4 ^a^	33.4 ± 0.3 ^c^	14.0 ± 2.2 ^a^	28.0 ± 0.1 ^a^	17.2 ± 0.7 ^b^
NaOH	26.1 ± 0.6 ^a^	11.0 ± 0.5 ^a^	30.2 ± 0.6 ^a^	13.6 ± 0.4 ^a^	28.1 ± 0.7 ^a^	15.9 ± 0.3 ^a^
Na_2_S_2_O_5_ + NaOH	27.0 ± 0.5 ^ab^	11.0 ± 0.3 ^a^	32.1 ± 0.6 ^b^	13.4 ± 0.4 ^a^	29.0 ± 0.7 ^a^	15.9 ± 0.5 ^a^

Data are means ± standard deviations, *n* = 3. Values in the same column with different superscript letters are significantly different (*p* < 0.05).

**Table 6:** Pasting parameters of starches.

	PV (mPa S)	HV (mPa S)	BV (mPa S)	FV (mPa S)	SV (mPa S)	PT (°C)
Ningzi 2						
H_2_O	5918 ± 85 ^b^	3167 ± 87 ^a^	2751 ± 72 ^b^	4103 ± 27 ^a^	936 ± 80 ^b^	78.2 ± 0.1 ^a^
Na_2_S_2_O_5_	5763 ± 50 ^a^	3092 ± 21 ^a^	2670 ± 52 ^b^	4053 ± 54 ^a^	961 ± 40 ^b^	78.5 ± 0.6 ^a^
NaOH	5804 ± 29 ^ab^	3308 ± 36 ^b^	2495 ± 12 ^a^	4027 ± 84 ^a^	719 ± 78 ^a^	80.8 ± 0.5 ^b^
Na_2_S_2_O_5_ + NaOH	5695 ± 66 ^a^	3213 ± 57 ^ab^	2482 ± 43 ^a^	4030 ± 40 ^a^	817 ± 43 ^ab^	81.6 ± 0.4 ^b^
Su 16						
H_2_O	6054 ± 55 ^a^	2309 ± 88 ^a^	3745 ± 66 ^b^	3466 ± 77 ^a^	1157 ± 63 ^c^	73.1 ± 0.5 ^a^
Na_2_S_2_O_5_	6143 ± 80 ^ab^	2322 ± 46 ^a^	3821 ± 46 ^b^	3625 ± 10 ^ab^	1303 ± 55 ^c^	73.0 ± 0.5 ^a^
NaOH	6134 ± 33 ^ab^	2730 ± 12 ^b^	3404 ± 26 ^a^	3711 ± 90 ^b^	980 ± 87 ^b^	75.2 ± 0.4 ^b^
Na_2_S_2_O_5_ + NaOH	6261 ± 62 ^b^	2762 ± 52 ^b^	3499 ± 48 ^a^	3487 ± 68 ^a^	725 ± 86 ^a^	75.0 ± 0.1 ^b^
Su 29						
H_2_O	5726 ± 10 ^a^	2328 ± 39 ^a^	3398 ± 29 ^b^	3268 ± 29 ^b^	940 ± 41 ^a^	74.7 ± 0.5 ^a^
Na_2_S_2_O_5_	5579 ± 62 ^a^	2297 ± 43 ^a^	3282 ± 42 ^a^	3133 ± 52 ^a^	836 ± 77 ^a^	74.8 ± 0.4 ^a^
NaOH	5714 ± 86 ^a^	2313 ± 59 ^a^	3401 ± 28 ^b^	3302 ± 50 ^b^	989 ± 58 ^ab^	77.6 ± 0.4 ^b^
Na_2_S_2_O_5_ + NaOH	5756 ± 98 ^a^	2245 ± 50 ^a^	3511 ± 68 ^c^	3352 ± 31 ^b^	1107 ± 79 ^b^	77.7 ± 0.4 ^b^

PV, peak viscosity; HV, hot viscosity; BV, breakdown viscosity (PV − HV); FV, final viscosity; SV, setback viscosity (FV − HV); PT, pasting temperature. Data are means ± standard deviations, *n* = 3. Values in the same column and variety with different superscript letters are significantly different (*p* < 0.05).

## References

[1] Bovell-Benjamin A.C. (2007). Sweet potato: A review of its past, present, and future role in human nutrition. Adv. Food Nutr. Res..

[2] Zhu F., Yang X., Cai Y.Z., Bertoft E., Corke H. (2011). Physicochemical properties of sweetpotato starch. Starch.

[3] Tang Y., Cai W., Xu B. (2015). Profiles of phenolics, carotenoids and antioxidative capacities of thermal processed white, yellow, orange and purple sweet potatoes grown in Guilin, China. Food Sci. Hum. Wellness.

[4] Wang S., Pan D., Lv X., Song X., Qiu Z., Huang C., Huang R., Chen W. (2016). Proteomic approach reveals that starch degradation contributes to anthocyanin accumulation in tuberous root of purple sweet potato. J. Proteom..

[5] Tanaka Y., Sasaki N., Ohmiya A. (2008). Biosynthesis of plant pigments: Anthocyanins, betalains and carotenoids. Plant J..

[6] Chen Z., Schols H.A., Voragen A.G.J. (2010). Physicochemical properties of starches obtained from three varieties of Chinese sweet potatoes. J. Food Sci..

[7] Genkina N.K., Takahiro N., Koltisheva G.I., Wasserman L.A., Tester R.F., Yuryev V.P. (2003). Effects of growth temperature on some structural properties of crystalline lamellae in starches extracted from sweet potatoes (Sunnyred and Ayamurasaki). Starch.

[8] Lai Y.C., Wang S.Y., Gao H.Y., Nguyen K.M., Nguyen C.H., Shih M.C., Lin K.H. (2016). Physicochemical properties of starches and expression and activity of starch biosynthesis-related genes in sweet potatoes. Food Chem..

[9] Kim J., Ren C., Shin M. (2013). Physicochemical properties of starch isolated from eight different varieties of Korean sweet potatoes. Starch.

[10] Lee B.H., Lee Y.T. (2016). Physicochemical and structural properties of different colored sweet potato starches. Starch.

[11] Osundahunsi O.F., Fagbemi T.N., Kesselman E., Shimoni E. (2003). Comparison of the physicochemical properties and pasting characteristics of flour and starch from red and white sweet potato cultivars. J. Agric. Food Chem..

[12] Senanayake S., Gunaratne A., Ranaweera K.K.D.S., Bamunuarachchi A. (2013). Physico-chemical properties of five cultivars of sweet potato (*Ipomea batatas* Lam) roots grown in Sri Lanka. Trop. Agric..

[13] Abegunde O.K., Mu T.H., Chen J.W., Deng F.M. (2013). Physicochemical characterization of sweet potato starches popularly used in Chinese starch industry. Food Hydrocoll..

[14] Londoño-Restrepo S.M., Rincón-Londoño N., Contreras-Padilla M., Millan-Malo B.M., Rodriguez-Garcia M.E. (2018). Morphological, structural, thermal, compositional, vibrational, and pasting characterization of white, yellow, and purple Arracacha lego-like starches and flours (*Arracacia xanthorrhiza*). Int. J. Biol. Macromol..

[15] Lin L., Guo D., Zhao L., Zhang X., Wang J., Zhang F., Wei C. (2016). Comparative structure of starches from high-amylose maize inbred lines and their hybrids. Food Hydrocoll..

[16] Correia P.R., Nunes M.C., Beirão-da-Costa M.L. (2013). The effect of starch isolation method on physical and functional properties of Portuguese nuts starches. II. Q. rotundifolia Lam. and Q. suber Lam. acorns starches. Food Hydrocoll..

[17] Estrada-León R.J., Moo-Huchin V.M., Ríos-Soberanis C.R., Betancur-Ancona D.B., May-Hernández L.H., Carrillo-Sánchez F.A., Cervantes-Uc J.M., Pérez-Pacheco E. (2016). The effect of isolation method on properties of parota (*Enterolobium cyclocarpum*) starch. Food Hydrocoll..

[18] Sun Q., Chu L., Xiong L., Si F. (2015). Effects of different isolation methods on the physicochemical properties of pea starch and textural properties of vermicelli. J. Food Sci. Technol..

[19] Baldwin P.M. (2001). Starch granule-associated proteins and polypeptides: A review. Starch.

[20] Debet M.R., Gidley M.J. (2006). Three classes of starch granule swelling: Influence of surface proteins and lipids. Carbohydr. Polym..

[21] Wei C., Qin F., Zhu L., Zhou W., Chen Y., Wang Y., Gu M., Liu Q. (2010). Microstructure and ultrastructure of high-amylose rice resistant starch granules modified by antisense RNA inhibition of starch branching enzyme. J. Agric. Food Chem..

[22] Zhang L., Zhao L., Bian X., Guo K., Zhou L., Wei C. (2018). Characterization and comparative study of starches from seven purple sweet potatoes. Food Hydrocoll..

[23] Lin L., Zhang Q., Zhang L., Wei C. (2017). Evaluation of the molecular structural parameters of normal rice starch and their relationships with its thermal and digestion properties. Molecules.

[24] Noda T., Takahata Y., Sato T., Ikoma H., Mochida H. (1996). Physicochemical properties of starches from purple and orange fleshed sweet potato roots at two levels of fertilizer. Starch.

[25] He W., Wei C. (2017). Progress in C-type starches from different plant sources. Food Hydrocoll..

[26] Waramboi J.G., Dennien S., Gidley M.J., Sopade P.A. (2011). Characterisation of sweetpotato from Papua New Guinea and Australia: Physicochemical, pasting and gelatinisation properties. Food Chem..

[27] Sevenou O., Hill S.E., Farhat I.A., Mitchell J.R. (2002). Organisation of the external region of the starch granule as determined by infrared spectroscopy. Int. J. Biol. Macromol..

[28] Warren F.J., Gidley M.J., Flanagan B.M. (2016). Infrared spectroscopy as a tool to characterize starch ordered structure—A joint FTIR-ATR, NMR, XRD and DSC study. Carbohydr. Polym..

[29] Blazek J., Gilbert E.P. (2011). Application of small-angle X-ray and neutron scattering techniques to the characterisation of starch structure: A review. Carbohydr. Polym..

[30] Sanderson J.S., Daniels R.D., Donald A.M., Blennow A., Engelsen S.B. (2006). Exploratory SAXS and HPAEC-PAD studies of starches from diverse plant genotypes. Carbohydr. Polym..

[31] Cai C., Cai J., Man J., Yang Y., Wang Z., Wei C. (2014). Allomorph distribution and granule structure of lotus rhizome C-type starch during gelatinization. Food Chem..

[32] Bogracheva T.Y., Morris V.J., Ring S.G., Hedley C.L. (1998). The granular structure of C-type pea starch and its role in gelatinization. Biopolymers.

[33] Li J.H., Vasanthan T., Hoover R., Rossnagel B.G. (2004). Starch from hull-less barley: V. In-vitro susceptibility of waxy, normal, and high-amylose starches towards hydrolysis by alpha-amylases and amyloglucosidase. Food Chem..

[34] Rincón-Londoño N., Vega-Rojas L.J., Contreras-Padilla M., Acosta-Osorio A.A., Rodriguez-Garcia M.E. (2016). Analysis of the pasting profile in corn starch: Structural, morphological, and thermal transformations, Part, I. Int. J. Biol. Macromol..

[35] Wang L., Wang Y.J. (2003). Rice starch isolation by neutral protease and high-intensity ultrasound. J. Cereal Sci..

[36] Rodriguez-Garcia M.E., Londoño-Restrepo S.M., Ramirez-Gutierrez C.F., Millan-Malo B. (2018). Effect of the crystal size on the X-ray diffraction patterns of isolated starches. arXiv.

[37] Wei C., Qin F., Zhou W., Yu H., Xu B., Chen C., Zhu L., Wang Y., Gu M., Liu Q. (2010). Granule structure and distribution of allomorphs in C-type high-amylose rice starch granule modified by antisense RNA inhibition of starch branching enzyme. J. Agric. Food Chem..

